# Diagnosis of Prostate Cancer and Prostatitis Using near Infra-Red Fluorescent AgInSe/ZnS Quantum Dots

**DOI:** 10.3390/ijms222212514

**Published:** 2021-11-19

**Authors:** Vuyelwa Ncapayi, Neethu Ninan, Thabang C. Lebepe, Sundararajan Parani, Aswathy Ravindran Girija, Richard Bright, Krasimir Vasilev, Rodney Maluleke, Ncediwe Tsolekile, Tetsuya Kodama, Oluwatobi S. Oluwafemi

**Affiliations:** 1 Department of Chemical Sciences, University of Johannesburg, P.O. Box 17011, Doornfontein, Johannesburg 2028, South Africa; vuyelwa.tito.ncapayi530@gmail.com (V.N.); calvyn.tl@gmail.com (T.C.L.); parani.sundararajan@gmail.com (S.P.); rodney.maluleke@gmail.com (R.M.); nncediwe@gmail.com (N.T.); 2 Centre for Nanomaterials Science Research, University of Johannesburg, P.O. Box 17011, Doornfontein, Johannesburg 2028, South Africa; 3 Academic Unit of STEM, University of South Australia, Mawson Lakes, SA 5095, Australia; neethun.ninan@gmail.com (N.N.); Richard.Bright@unisa.edu.au (R.B.); Krasimir.Vasilev@unisa.edu.au (K.V.); 4 Future Industries Institute, University of South Australia, Mawson Lakes, SA 5095, Australia; aswathy.ravindrangirija@tekcyte.com; 5 Department of Biomedical Engineering, Graduate School of Biomedical Engineering, Tohoku University, 4-1 Seiryo-machi, Aoba-ku, Sendai 980-8575, Japan; kodama@tohoku.ac.jp

**Keywords:** near infra-red quantum dots, bioimaging, prostate cancer, Gram-positive bacteria, Gram-negative bacteria

## Abstract

The link between the microbiome and cancer has led researchers to search for a potential probe for intracellular targeting of bacteria and cancer. Herein, we developed near infrared-emitting ternary AgInSe/ZnS quantum dots (QDs) for dual bacterial and cancer imaging. Briefly, water-soluble AgInSe/ZnS QDs were synthesized in a commercial kitchen pressure cooker. The as-synthesized QDs exhibited a spherical shape with a particle diameter of 4.5 ± 0.5 nm, and they were brightly fluorescent with a photoluminescence maximum at 705 nm. The QDs showed low toxicity against mouse mammary carcinoma (FM3A-Luc), mouse colon carcinoma (C26), malignant fibrous histiocytoma-like (KM-Luc/GFP) and prostate cancer cells, a greater number of accumulations in *Staphylococcus aureus*, and good cellular uptake in prostate cancer cells. This work is an excellent step towards using ternary QDs for diagnostic and guided therapy for prostate cancer.

## 1. Introduction

The microbiome comprises both helpful and potentially harmful microbes that have achieved wide attention since 1676 when bacteria and protozoans were first discovered [1]. The intestinal microbiome consists of countless bacteria that act as a protective shield for the body against foreign microbes and that assist in metabolizing complex carbohydrates that are unprocessed by the human body [2]. The implication of this symbiotic association took a paradigm alteration in the late 19th century when *Streptococcus* bacteria was detected in two patients with sarcoma [3]. Since then, strong links have been developed between the microbiome and cancer, proving their negative impacts. The microbiome was found to impact every stage of cancer from initiation to progression and metastasis. Cancer patients are at a high risk of bacterial infections as bacteria can enter the cancer cells through the leaky vasculature, thrive in hypoxic nutrient-rich regions of cancer cells, and become pathogenic when the immune system is down [4]. The exquisite research by Nobel Laureates Warren and Marshall on the role of *Helicobacter pylori* in gastric cancer further demonstrates the bacteria–cancer interactions [3].

Prostate cancer is the second most common cause of cancer in men that shows wide inter-patient genetic heterogeneity [5]. Prostatic inflammation is a serious risk factor in prostate cancer development, caused by pathogenic microorganisms [6]. The causative pathogens in prostatitis include Gram-negative bacteria (*Escherichia coli*, *Pseudomonas*, *Klebsiella*, *Proteus*, etc.) and Gram-positive bacteria (*Streptococcus*, *Staphylococcus*, *Enterococci*, *Corynebacterium*, etc.). The surgical approaches for prostate cancer include brachytherapy, radical prostatectomy, external beam radiation therapy, etc. In radical prostatectomy, the whole prostate gland is removed, and the urethra and bladder are sutured, leading to severe complications [7]. Nerve-sparing surgery can minimize these complications but often suffer from the risk of recurrence due to the presence of positive surgical margins [8]. Again, cancer patients are treated with a huge dosage of chemo drugs with potential side effects. The presence of bacteria inside the tumor can impart resistance against cancer drugs due to their intrinsic ability to cleave the chemo drugs to inactive metabolites. This will further complicate the scenario as immunosuppressed cancer patients will be given more antibiotics and become antibiotic-resistant. Therefore, an expedient method to detect and locate prostate cancerous lesions and infections in tissues during operation would be extremely useful, which will enable medical practitioners to analyze the complexity of the disease and decide an effective way to prevent the spread of cancer.

Fluorescent probes with near-infra-red (NIR) emission are excellent tools for biomedical imaging due to their high fluorescence intensity, low cytotoxicity, and deep tissue penetration [8,9]. Quantum dots (QDs) are one of such fluorescent probes used for imaging due to their exceptional photochemical stability, size-tunable luminescence, and resistance to chemical degradation. These unique characteristics make them more useful than fluorescent proteins and organic dyes [10,11]. However, their progress as imaging tools in diagnostics has been hampered by their cytotoxicity and the challenges associated with the synthetic method and the type of precursors used. Various approaches have been reported for the development of QDs with little or no toxicity on the normal cell lines. The approaches have focused on proper passivation, hydrophilicity, and high fluorescence stability in various mediums to reduce their toxicity [12]. Our group has recently reported the synthesis of non-cadmium-based toxic-free and highly biocompatible CuInS/ZnS and AgInSe/ZnSe QDs that emit at NIR wavelengths [13]. Herein, we developed a facile and potent strategy for the synthesis of AgInSe/ZnS core-shell QDs as cancer–bacterial dual imaging tools for prostate cancer and prostatitis, which can aid in the development of an effective therapeutic method.

## 2. Results

### 2.1. Physicochemical Characterization

**Figure 1:**
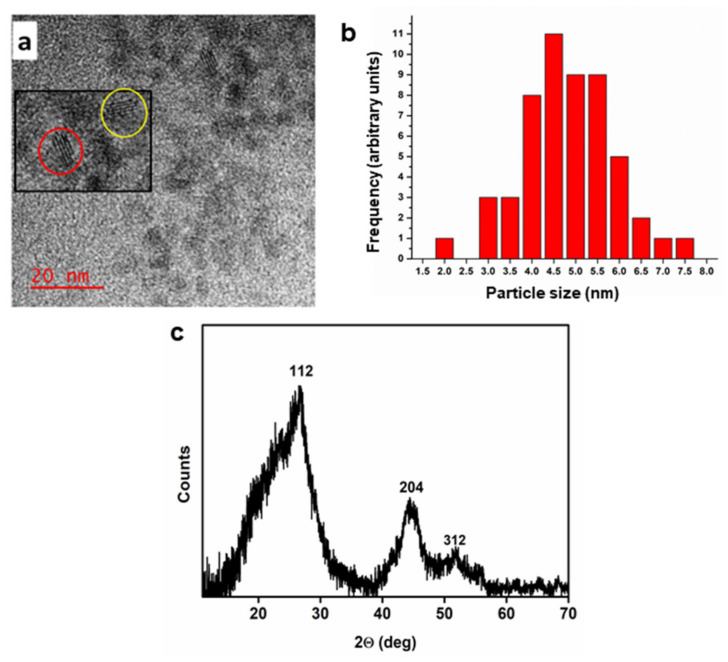
(**a**) TEM micrograph, and (**b**) particle size distribution (**c**) XRD of the as synthesized AgInSe/ZnS QDs.

**Figure 2:**
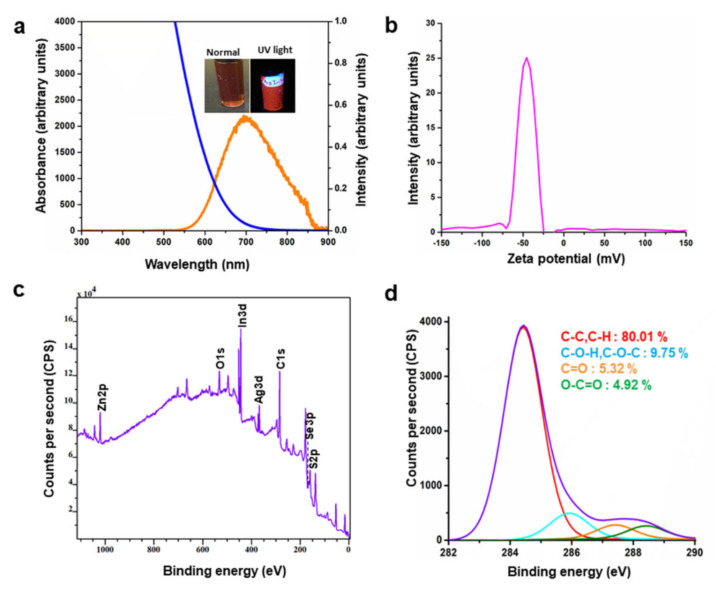
(**a**) Photoluminescence & UV absorption spectra (with images insert under normal and UV light at 365 nm), (**b**) Zeta potential spectra, (**c**) XPS survey spectra, and (**d**) high-resolution C1s spectra of AgInSe/ZnS QDs.

QDs have played a pivotal role in biological applications due to their particle size and good optical properties. The size of QDs (0–10 nm) makes them ideal for drug delivery as they can penetrate through the cells. In addition, their NIR emitting ability with high fluorescence intensity makes them suitable as an imaging probe in diagnostics. Thus, the optical, as well as the structural, properties of AgInSe/ZnS QDs were studied. The as-synthesized and purified QD solutions were stable for a long time (>6 months) with no visible aggregation. The typical TEM micrograph of the as-synthesized AgInSe/ZnS QDs is shown in Figure 1. The micrograph shows that they are small and nearly spherical. The particle size ranges from 2.2 nm to 7.5 nm with an average particle diameter of 4.5 nm ± 0.5 nm. The presence of a lattice fringe (Figure 1a inset) confirmed the crystalline nature of the as-synthesized QDs. The diffractogram proved the crystallinity of the QDs and showed broad peaks close to the tetragonal phase of AgInSe_2_ (JCPDS 75-0118), (Figure 1c). The absorption spectrum (Figure 2a) shows no distinct absorption maximum, which is characteristic of ternary chalcopyrite QDs. The luminescence spectrum shows a maximum emission peak at 705 nm. The quantum yield was 10.16%, which is within the range of what has been reported by Kang et al. [14]. Furthermore, the QDs displayed an orange color under normal light and emitted in the red region when irradiated at 365 nm under a UV lamp (Figure 2a inset). The high stability of the QDs was confirmed by the Zeta potential analysis, which gave a value of −45.4 mV (Figure 2b). The XPS survey analysis (Figure 2c) indicates the presence of Ag3d, In3d, Se3p, Zn2p, and S2p, which are prominent elements of the as-synthesized AgInSe/ZnS core/shell quantum QDs, while the C and O signals were due to the presence of a capping agent (citrate and thioglycolic acid) on the QDs surface. The deconvoluted carbon spectra (Figure 2d) showed 80.01% C–C, 9.75% C–OH, 5.32% C=O, and 4.92% O–C=O, which are characteristic functional groups found in the dual stabilizers.

### 2.2. Cytotoxicity

The MTT assay for AgInSe/ZnS QDs was carried out on malignant fibrous histiocytoma-like KM-Luc/GFP cells, expressing a fusion of the luciferase and enhanced-green fluorescent protein genes, (KM-Luc/GFP), C3H/He mouse mammary carcinoma cells (FM3A-Luc), and mouse colon carcinoma (C26) with n = 3 in 96-well plates, as described in the experimental section. The cell survival ratio was determined by the following equation:

% Cell viability = (Ax/A0) × 100%.

where Ax and A0 represent the absorbance of the QDs treated cells and the absorbance of the control, respectively.

**Figure 3:**
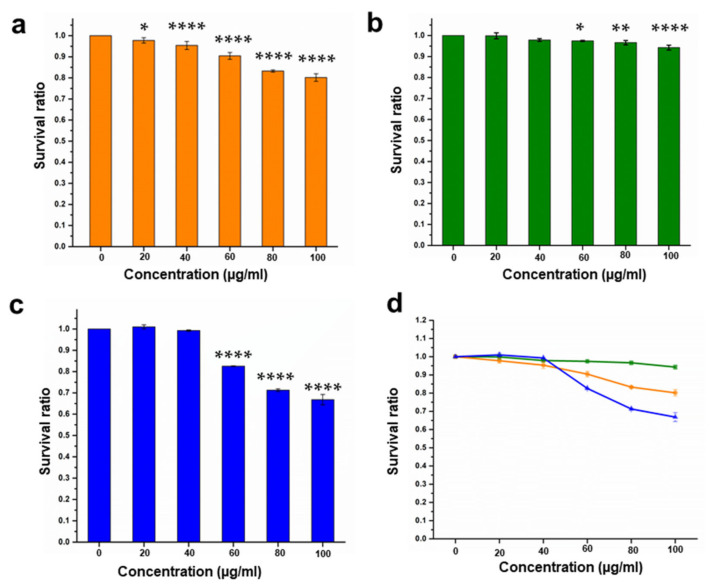
Cytotoxicity of AgInSe/ZnS QDs against (**a**) FM3A-Luc, (**b**) C26, (**c**) KM-Luc GFP, and (**d**) summary at different concentrations. Statistical significance was assessed by one-way ANOVA with Tukey’s multiple comparison test for * *p* < 0.1, ** *p* < 0.01, and **** *p* < 0.0001, compared to control.

As demonstrated in Figure 3a–d, the QDs behave differently in all the three cancer cell lines without reaching the 50% lethal concentration (LC50), which is used as the measure of acute toxicity in medical treatment when the MTT assay protocol is employed. It was observed that the survival ratio is concentration- and cell-type-dependent. The survival ratios of 94%, 80%, and 67% were obtained for the C26, FM3A-Luc, and KM-Luc/GFP cell lines at 100 µg/mL, respectively, indicating that the as-prepared QDs possessed good cell viability.

### 2.3. Biofilm Imaging

**Figure 4:**
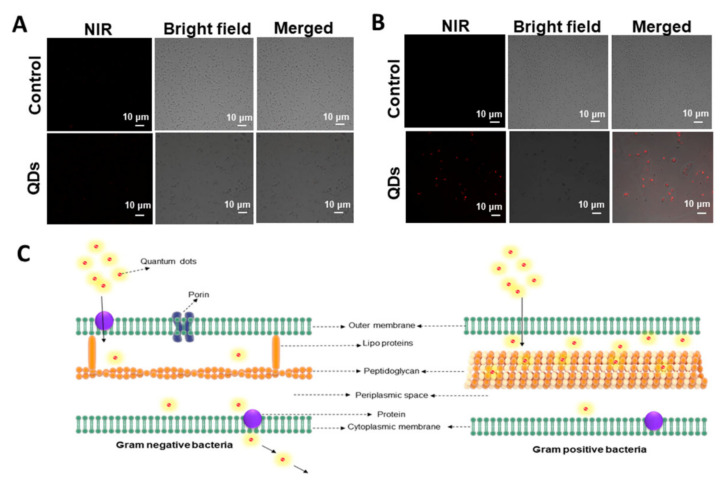
Confocal microscopy images showing the uptake of AgInSe/ZnS QDs in (**A**) *Staphylococcus aureus* (Gram-positive bacteria) and (**B**) *Escherichia coli* (Gram-negative bacteria). (**C**) A possible mechanism for the differential uptake of AgInSe/ZnS QDs in Gram-positive and Gram-negative bacteria.

**Figure 5:**
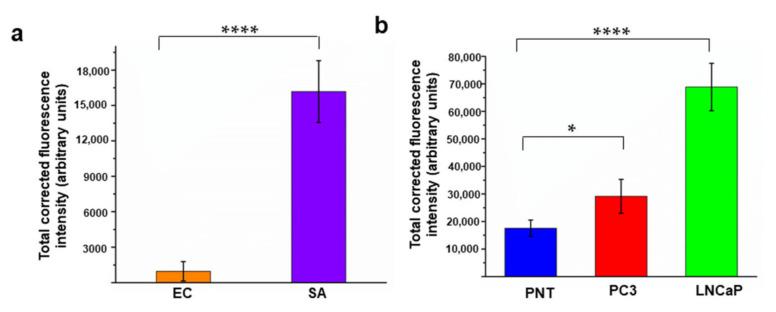
Total corrected fluorescence intensity of AgInSe/ZnS QDs in (**a**) Gram-negative *Escherichia coli* (EC) and Gram-positive *Staphylococcus aureus* (SA); (**b**) prostate cells PNT, PC3, and LNCaP. Statistical significance was assessed by one-way ANOVA with Dunnett’s multiple comparison test for * *p* < 0.1 and **** *p* < 0.0001.

The QDs have shown great potential as multiwavelength fluorescent labels for imaging cells [15]. The causative pathogens in prostatitis include both Gram-positive and Gram-negative bacteria. To verify whether the QDs can penetrate and stain the biofilms, confocal fluorescence imaging experiments were conducted on *S. aureus* (Gram-positive) and *E. coli* (Gram-negative) biofilms treated separately with the QDs. Figure 4 showed that the small size of QDs enabled them to penetrate the bacterial biofilms, which allowed significant fluorescence enhancement with greater imaging. Another interesting feature to note is that the QDs could selectively stain Gram-positive and Gram-negative bacteria. There were a greater number of QDs in Gram-positive bacteria than Gram-negative bacteria, which might be attributed to the strong interaction of the negative surface charge of the QDs with Gram-positive bacteria via strong electrostatic attraction [16]. Another possible reason can be the thicker peptidoglycan wall of Gram-positive bacteria that can retain the QDs. On the other hand, the thinner peptidoglycan wall of Gram-negative bacteria allows the QDs to be easily removed from the cells. This was again supported by the fluorescence intensities evaluated using ImageJ software (Figure 5a). The fluorescence intensities of QDs were higher in *S. aureus* compared to *E. coli*. When compared with the conventional Gram-staining method, the QD-based Gram-type differentiation approach is accurate, fast, and easy to operate.

### 2.4. Cancer Cell Imaging

**Figure 6:**
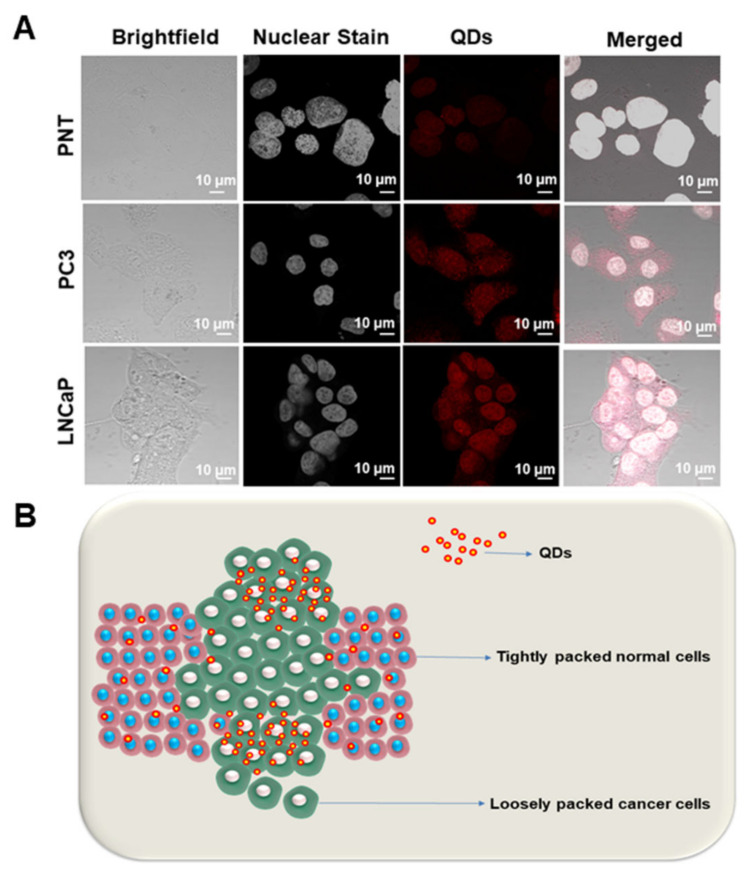
(**A**) Confocal microscopy images showing the uptake of AgInSe/ZnS QDs in prostate normal cells (PNT), prostate cancer cells (PC3), and human prostate adenocarcinoma cells (LNCaP). (**B**) Possible mechanism for the differential uptake of QDs in cancer and normal cells.

In vitro fluorescent imaging capabilities of QDs were investigated by confocal fluorescent imaging. The internalization of the QDs was studied on normal pancreatic cells (PNT) and pancreatic cancer cells (PC3 and LNCaP cells). As evident from Figure 6, the cellular uptake of the QDs was highest in the cancer cells (LNCaP and PC3), whereas their uptake was negligible in the case of normal PNT cells. The fluorescence intensity was quantified using ImageJ software, which further supports our observation. QDs exhibited higher fluorescence intensities in cancer cells (LNCaP and PC3) compared to normal PNT cells (Figure 5b). QDs tend to accumulate more in tumor tissue than normal tissue due to enhanced permeability and the retention effect, which allows them to diffuse passively through the leaky vasculature and accumulate inside the cells [17]. The loss of lipid symmetry in cancer cells also allows the preferential uptake of QDs [18]. As evident, the quantum dots were well dispersed throughout the entire cytoplasm of each cell, and there were no visible signs of aggregation. A distinct striking pattern of fluorescence intensity was observed in cells exposed to QDs (Figure 5a). The high fluorescence intensity of the QDs suggests the proper passivation and photostability of the QDs, thereby reducing the quenching effect.

## 3. Materials and Methods

### 3.1. Materials

Silver nitrate (AgNO_3_), indium nitrate (In(C_2_H_3_O_2_)_3_), sodium selenite (Na_2_SeO_3_), thioglycolic acid (TGA), tri-sodium citrate (CT), sodium borohydride (NaBH_4_), zinc acetate dihydrate (ZnC₄H₆O₄), and ammonium hydroxide (NH_4_OH) were purchased from Sigma Aldrich (Kempton Park, South Africa). All chemicals were of analytical grade and used as received. Phosphate-buffered saline (PBS) and Dulbecco’s Modified Eagle’s Medium (DMEM) were purchased from Sigma Aldrich (St. Louis, MO, USA). Penicillin and Streptomycin were procured from Life Technologies (Carlsbad, CA, USA). Fetal bovine serum (FBS) was bought from Thermo Scientific (Waltham, MA, USA). Tryptone Soy Broth (TSB) was obtained from Oxoid (Thebarton, Australia).

### 3.2. Synthesis of AgInSe/ZnS QDs

**Scheme 1:**
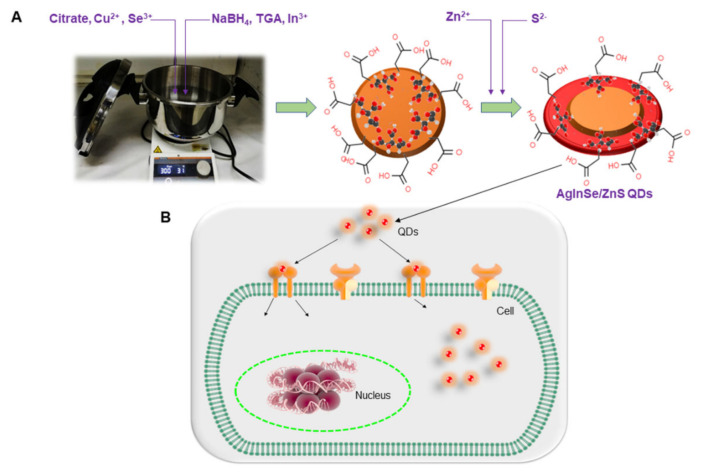
(**A**) Schematic illustration to show the synthesis of AgInSe/ZnS QDs and (**B**) application of AgInSe/ZnS QDs in cell tracking or imaging.

The synthesis of AgInSe/ZnS core/shell QDs was carried out in a commercial kitchen pressure cooker based on our recent method [19], with some modifications as shown in Scheme 1. Briefly, 0.1306 g (0.77 mM) silver nitrate was dissolved in 1500 mL of deionized water in a kitchen pressure cooker. The indium stock solution was prepared by dissolving 0.79063 g (3.08 mmol) indium acetate in 5 mL water, 3.2 mL TGA, and 3 mL ammonium hydroxide. Sodium tri-citrate (25.1132 g, 9.73 mol) was weighed and transferred directly to the pressure cooker, while selenium stock solutions (20 mL) prepared by dissolving 0.6646 g (3.85 mM) of sodium selenite in water were also transferred to the pressure cooker. The pressure cooker was then heated at 120 °C for the synthesis of AgInSe core QDs. After 1.5 h, the solution was cooled naturally to 80 °C, then zinc (3.85 mmol Zinc acetate) and sulphur (3.85 mmol thiourea) precursors were added, and the reaction was continued at this temperature for another 1 h to prepare AgInSe/ZnS QDs. The as-synthesized QDs were then precipitated by ethanol and collected via centrifugation, washed with ethanol, and dried at ambient conditions.

### 3.3. Characterization

The optical properties of the freshly prepared and purified QDs solution were evaluated by using ultraviolet–visible spectrophotometry (UV–vis) (Perkin Elmer UV–Vis Lambda 25 spectrometer, Beaconsfield, UK) and a photoluminescence (PL) (RF-6000, Shimadzu, Kyoto, Japan) spectrofluorometer. The charge of the QDs was measured using a Zeta potential analyzer (photal ELS-Z2MH, Otsuka Electronics Co., Ltd., Osaka, Japan). The particle diameter and crystallinity of the QDs were evaluated using the JEOL JEM-3010 (Tokyo, Japan) electron microscope operating at 200 kV by dropping the QDs solution in a copper-coated grid and allowing it to air dry before capturing the images at different resolutions. The crystallinity of the QDs were determined using a Bruker D8 Advance X-ray (Bruker GmbH, Karlsruhe, Germany) diffractometer using monochromatic Cu Kα 1 radiation (γ = 1.54 Å) at the diffraction angle range of 10° and 90°. The elemental composition of the QDs was evaluated using a Kratos AXIS Ultra DLD (Kratos Analytical, Manchester, UK) spectrometer provided with a monochromatic Al source set at 15 keV and 15 mA. Survey spectra were obtained over a 0−1100 eV range with a pass energy of 160 eV, and high-resolution spectra were collected using a 20-eV pass energy. Casa XPS software (Version 2.3.16, Casa Software Ltd., Teignmouth, UK), was used to process the obtained spectra, and all binding energies were referenced to the carbon peak at 285.0 eV.

### 3.4. Cytotoxicity

The cytotoxicity of the as-synthesized AgInSe/ZnS QDs was evaluated against three cell lines, C3H/He mouse mammary carcinoma (FM3A-Luc), mouse colon carcinoma (C26), and malignant fibrous histiocytoma (KM-Luc/GFP) using the MTT assay protocol. All cells were obtained from the Cell Resource Center for Biomedical Research, Institute of Development, Aging and Cancer, Tohoku University, Sendai, Japan. The MTT assay kit contains water-soluble tetrazolium salts that can be reduced to purple formazan [20]. The cells were cultured and maintained at 37 °C in a 5% CO_2_ incubator using an appropriate medium and then seeded at the concentration of 1.0 × 10^4^ cells/mL in a 96-well plate followed by impregnation with different QDs concentrations (0–100 µg/mL) and incubation for 24 h before the addition of 10 µL of MTT solution. The solution was incubated for another 1 h, and its absorbance was measured using a microplate absorbance reader. The values were later converted to a % survival ratio. All the experiments were done in triplicate (n = 3).

### 3.5. Biofilm Imaging

*Staphylococcus aureus* (ATCC 25923) and *Escherichia coli* (ATCC 25922) cultures were inoculated in tryptone soy broth (TSB), incubated overnight at 37 °C, and allowed to reach the mid-log phase. The bacterial suspension was then diluted to 1 × 10^7^ CFU/mL using sterile TSB, which was added to wells containing glass coverslips, and incubated for 20 h. After incubation, 50 µL of QDs (1 mg/mL) were added to these wells and incubated for a further 3 h at 37 °C. The cellular uptake was visualized using an Olympus FV3000 confocal laser scanning microscope (Olympus, Tokyo, Japan). The excitation and emission wavelengths for QDs were 450 nm and 859 nm, respectively. Single-plane fluorescence micrographs were acquired from randomly chosen areas on the glass surface in triplicates. The fluorescence intensity was later quantified using ImageJ software (Version 1.51, National Institute of Heath, Maryland, MD, USA) and plotted.

### 3.6. Prostate Cancer Cell Imaging

The normal prostate cell line (PNT), the prostate cancer cell line (PC3), and human prostate adenocarcinoma cells (LNCaP) were cultured and maintained separately using DMEM supplemented with 10% of FBS in a 5% CO_2_ incubator at 37 °C. The cells were seeded into a 33-mm glass base dish for imaging at a cell concentration of 1.5 × 10^4^ cells/plate for 24 h. Twenty µL of the QDs (1 mg/mL) were added to the cells and incubated for another 2 h to observe passive absorption of QDs by cancer cells. After 2 h, unabsorbed QDs were removed by washing with cold PBS several times. The cells were fixed using 4% paraformaldehyde for 30 min at room temperature. This was followed by the addition of 0.5% Triton X 100 (Sigma-Aldrich, St. Louis, MO, USA) for 15 min in an ice bath. Samples were washed three times with PBS and incubated with the nuclear counterstain NucBlue (Life Technologies, Carlsbad, CA, USA) for 15 min. The staining solution was removed by PBS washing and stored at 4 °C. The fluorescent images were acquired using an Olympus FV3000 confocal laser scanning microscope using bandpass excitation and emission filters at 450 nm and 859 nm, respectively. The fluorescence intensity was later quantified using ImageJ software and plotted.

## 4. Conclusions

In summary, water-soluble NIR-emitting AgInSe/ZnS QDs were synthesized using a commercial kitchen pressure cooker. The as-synthesized QDs were nearly spherical with an average particle diameter of 4.5 ± 0.5 nm and high colloidal stability. The MTT assay revealed that the QDs were biocompatible to FM3A-Luc, C26, and KM-Luc GFP cells even at high concentrations. The bioimaging results showed that the AgInSe/ZnS QDs more effectively stained *S. aureus* (Gram-positive bacteria) than *E. coli* (Gram-negative bacteria) due to its negative charge. In addition, the QDs were able to selectively target prostate cancer cells with high cellular uptake than normal prostate cells. Thus, the synthesized AgInSe/ZnS QDs produced could be employed as an imaging probe for guided diagnostic and therapy.

## Data Availability

Data available on request due to restrictions.
